# Fabrication of Diaminodiphenylmethane Modified Ammonium Polyphosphate to Remarkably Reduce the Fire Hazard of Epoxy Resins

**DOI:** 10.3390/polym13193221

**Published:** 2021-09-23

**Authors:** Feiyue Wang, Jiahao Liao, Long Yan, Hui Liu

**Affiliations:** Institute of Disaster Prevention Science and Safety Technology, School of Civil Engineering, Central South University, Changsha 410075, China; wfyhn@163.com (F.W.); 194812294@csu.edu.cn (J.L.); lhui0421@163.com (H.L.)

**Keywords:** epoxy resin, diaminodiphenylmethane, ammonium polyphosphate, flame retardancy, smoke suppression, mechanical property

## Abstract

A novel diaminodiphenylmethane (DDM) modified ammonium polyphosphate (APP) flame retardant, DDP, was successfully synthesized via ion-exchange reaction. DDP was introduced into epoxy resins (EPs) to reduce flammability. A comparable level of DDP exerts better flame-retardant and smoke suppression efficiencies in EP than APP. An EP blend containing 15 wt% DDP displays a limiting oxygen index (LOI) value of 37.1% and a UL 94 V-0 rating, and further exhibits a 32.3% reduction in total heat release and a 48.0% reduction in total smoke production compared with pure EP. The presence of DDP greatly facilitates char formation during combustion, and the char mass from thermal decomposition of an EP blend is 37.8% smaller than that of an EP blend containing 15 wt% DDP at 800 °C. The incorporation of DDP into EP blends has a smaller impact on the glass transition temperature and tensile strength than those of a comparable level of APP. This reflects the better compatibility of DDP with the EP matrix compared with that for APP.

## 1. Introduction

Epoxy resin (EP) has been widely applied in the production of electronic appliances, in machinery, in chemical anti-corrosion formulations, in aerospace, and by defense and national economy departments because of its excellent physical properties, chemical resistance, adhesive characteristics, and corrosion resistance [1]. However, epoxy resin is highly flammable and undergoes combustion with the generation of large amounts of smoke. These combustion characteristics represent a threat to human life and limit the use of epoxy resin in many areas. Therefore, effective means of reducing the flammability of epoxy resin are required [2].

Flame retardant treatment of epoxy resin is required to reduce flammability; traditionally, organohalogen flame retardants have been used for this purpose. However, these materials decompose in the polymer undergoing thermal degradation to generate volatile, very toxic dioxins [3,4]. Further, when items containing these flame retardants are discarded in a landfill, they are leached into the environment in which they are stable, persist, bioaccumulate, and enter the human food chain. Human exposure leads to the development of a variety of diseases. Furthermore, organohalogen tends to bioaccumulation, which pose threats to environment, wildlife, and human body [5,6]. For this reason, these compounds are being removed from the market. Metal hydroxide flame retardants absorb large amounts of heat during decomposition to release non-combustible gases that dilute the fuel load in the combustion zone [7]. However, the presence of very large amounts (40~60 wt%) of these materials is required to achieve flame retardancy. At this level of loading, the mechanical properties of the matrix resin are so compromised that the material is unsuitable for many applications. Intumescent flame retardants have received much affection. In general, these materials display low toxicity and good flame retardant efficiency. They function by promoting a char layer at the surface of the polymer [8,9]. This char layer serves as an insulation barrier to inhibit heat feedback from the combustion zone and limit the formation of volatile fuel fragments.

In general, intumescent flame retardants can be classified into additive flame retardants and reactive flame retardants. The additive intumescent flame retardant means adding flame retardants by physical blending into epoxy resin, which has poor compatibility with the unsustainable flame-retardant efficiency. The reactive intumescent flame retardant combines the surface group of flame retardant with the matrix through chemical bonds, which permits the retention of the flame retardant effect permanently. It has little influence on the original mechanical and thermal properties [10]. Ammonium polyphosphate (APP) has been widely used as an additive flame retardant in polymers thanks to its good char promotion and flame retardant properties. However, the compatibility between APP and the polymer matrix is poor, which leads to problems of precipitation, moisture absorption, and low flame retardant efficiency [11,12]. Improvement of the flame retardant efficiency and compatibility of APP with polymer materials has been variously reported. In past studies, a substance containing an amine group [13,14], organometallic framework [15], and bio-based resources [16] was used to modify APP. Among them, amine-containing modified APP has the characteristics of simple synthesis and good comprehensive performance, which provides a scheme for the synthesis of a high-efficient flame retardant with super performance. However, no study has reported that DDM could improve the flame retardant effect and compatibility of APP in epoxy resin.

In this study, DDM was grafted onto the surface of APP via an ion exchange method to obtain DDP, and DDP was characterized using Fourier transform infrared (FTIR) spectroscopy, nuclear magnetic resonance (^1^H NMR, ^31^P NMR), scanning electron microscopy (SEM)–energy-dispersive X-ray, and thermo-gravimetric (TG) analyses. Then, the obtained DDP flame retardant was incorporated into epoxy resin to prepare flame-retarded EP blends. The flame retardant performance, thermal performance, and influence of mechanical properties of EP blends with DDP were studied in detail.

## 2. Materials and Methods

### 2.1. Materials

Ammonium polyphosphate (form II) with an average polymerization of 1000 was obtained from Hangzhou JLS Flame Retardants Chemical Co., Ltd. (Hangzhou, China). Epoxy resin (E-44) was purchased from Zhenjiang Danbao Resin Co., Ltd. (Zhenjiang, China). DDM was provided by Changzhou Runxiang Chemical Co., Ltd. (Changzhou, China). Ethanol was provided by Tianjin Hengxing chemical reagent manufacturing Co., Ltd. (Tianjin, China). The above reagents were used as received.

### 2.2. Synthesis of DDP Flame Retardant

**Figure 1:**
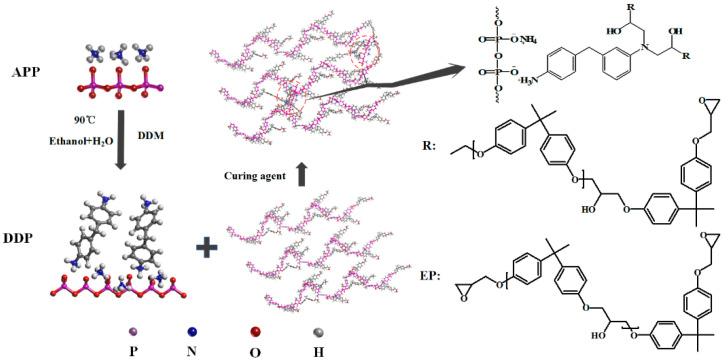
Preparation of DDP and its flame-retarded EP blends.

In this study, 200 mL ethanol and 10 mL water were poured into a three-necked flask equipped with a magnetic stirrer, and the mixed solution was heated to 60 °C for 10 min. Then, 10 g DDM was injected into above mixed solution and stirred for about 10 min. As DDM completely dissolved, 20 g APP powder was added into the flask slowly, and the mixture was heated up to 60 °C for 4 h. After the reaction, the mixed solution was cooled to room temperature and then filtered to obtain a yellowish solid. The resulting solid was washed with ethanol three times and then dried at 60 °C for 12 h to obtain DDP, and the yield of DDP is 78.3%. The synthesis process of DDP is shown in Figure 1.

### 2.3. Preparation of Flame-Retarded EP Blends

The preparation process of flame-retarded EP blends is presented in Figure 1, and the composition of the EP blends is shown in Table 1. Firstly, epoxy resin and DDP were mixed and stirred at a speed rate of 400 rpm/min at 80 °C for 10 min, and then stirred at 90 °C for 5 min. Secondly, the above mixture was mixed with DDM with stirring for 2 min at 400 rpm/min and then quickly poured into the preheated PTFE mold. Finally, the mold was put in an electric blast drying oven for 2 h at 120 °C and cured at 150 °C for 1 h.

### 2.4. Characterization and Measurements

FTIR with a wavenumber range 4000–500 cm^−1^ was obtained using i CAN 9 infrared spectrometers (Tianjin Energy Spectrum Technology Co., Ltd.) by the KBr disk method. ^1^H NMR and ^31^P NMR measurements were taken on AVANCE400 Bruker at room temperature using DMSO-d_6_ as solvent.

The microcosmic morphologies of DDP and char residues were observed by SEM analyzer (TESCAN MIRA3 LMU, Brno, Czech Republic) with a voltage of 20 kV. EDS was used to investigate the elemental content and element distribution of the samples by an X-Max20 X-ray probe (Oxford Instruments, Abingdon Oxon, UK).

TG was carried out on TGA/SD-TA851e thermal gravimetric analyzer (Mettler Toledo International Trading Co., Ltd., Shanghai, China). About 2~5 mg samples were heated from room temperature to 800 °C under air purges. The theoretical amount of char residue (*W*_cal_) was obtained from Formula (1).

(1) $$W_{\mathrm{cal}}(T)=\sum_{i=1}^{n}\;\chi_{i}W_{i}(T)$$

where *χ*_i_ represents the content of compound i, and *W*_i_ represents the experimental char residual weight of compound i in the TG test.

DSCs were carried out on DSC823e (Mettler Toledo International Trading Co., Ltd., Shanghai, China). The sample was heated at a heating rate of 10 °C min^−1^ from 25 °C to 220 °C under 50 mL min^−1^ nitrogen atmosphere.

LOI values were obtained on an HC-2CZ oxygen index meter (Nanjing Shangyuan Analytical Instrument Co. Ltd., Nanjing, China). The test procedure is according to ASTM D2863-19” Standard Test Method for Measuring the Minimum Oxygen Concentration to Support Candle-Like Combustion of Plastics (Oxygen Index)”, and the sample sizes for testing are 130 × 6.5 × 3.2 mm^3^.

UL94 test rating was acquired on a JL8333-3 Foam horizontal combustion tester (Nanjing Jionglei Instrument Equipment Co., Ltd., Nanjing, China) according to the UL94-2016 “standard for safety: Tests for Flammability of Plastic Materials for Parts in Devices and Appliances”, and the specimens used are 130 × 3 × 3.2 mm^3^.

Cone calorimeter (FTT 0007) was used to characterize the flammability of the samples according to ISO 5660-1 “Reaction-to-fire tests—Heat release, smoke production, and mass loss rate—Part 1: Heat release rate (cone calorimeter method) and smoke production rate (dynamic measurement)”. All the samples were irradiated horizontally at a heat flux of 50 kW m^−1^. The sample size used for detection is 100 × 100 × 3.5 mm^3^, which was horizontally mounted on aluminum foil.

Tensile tests were measured with a WDW-10D microcomputer controlled electronic universal testing machine (Jinan Hengsi shengda Instrument Co., Ltd., Jinan, China). The test speed was set to 5 mm/min, and the sample size is type I standard sample according to GB/T 1040.2-2006 “Plastics-Determination of tensile properties-Part 2: Test conditions for moulding and extrusion plastics”.

## 3. Results

### 3.1. Preparation of DDP

**Figure 2:**
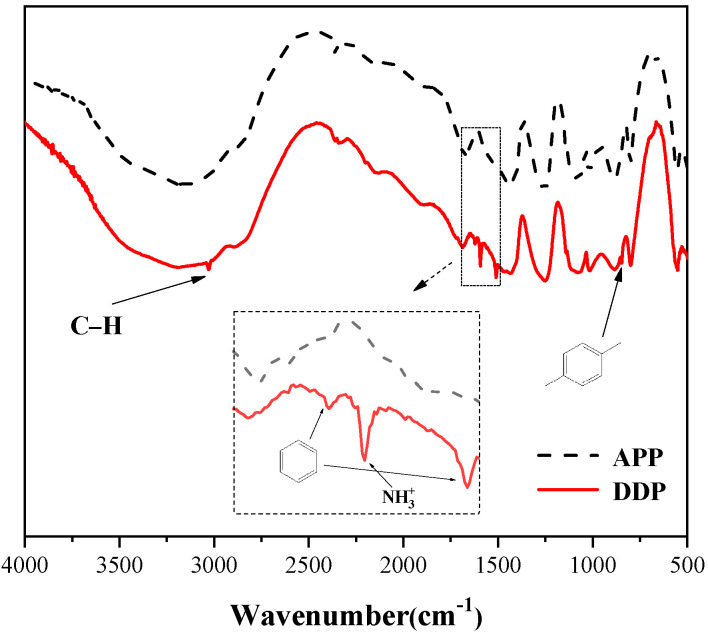
FTIR spectra of DDP and APP.

FTIR spectra of APP and DDP are presented in Figure 2. As shown in Figure 2, DDP has five new characteristic absorption peaks at 3027 cm^−1^, 1621 cm^−1^, 1592 cm^−1^, 1508 cm^−1^, and 846 cm^−1^ compared with APP. The characteristic peak at 3027 cm^−1^ corresponds to the stretching vibration peak of the C–H bond on benzene ring [17], and the peaks at 1614 cm^−1^ and 1508 cm^−1^ correspond to the characteristic stretching absorption of skeleton vibration of the benzene ring. In addition, the peak at 846 cm^−1^ and 1592 cm^−1^ corresponds to the 1,4 substituents on the surface of the benzene ring in DDM and the characteristic absorption peak of the –NH_3_^+^ structure. The peak at 1592 cm^−1^ is the characteristic absorption peak of the –NH^3+^ structure, exhibiting the existence of the reaction between DDM and APP [13]. It can be seen from the FTIR results that DDP has both functional groups of APP and DDP, which can preliminarily indicate the successful synthesis of DDP flame retardant.

**Figure 3:**
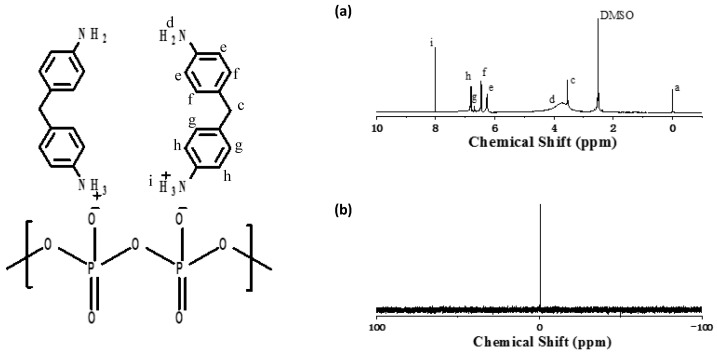
^1^H NMR (**a**) and ^31^P NMR (**b**) spectra of DDP.

^1^H NMR and ^31^P NMR spectra of DDP are presented in Figure 3 in the ^1^H NMR spectrum of DDP. Moreover, the chemical shift at 8.2 ppm corresponds to the amino active hydrogen atom at the end of DDP [18]; the chemical shift in the range of 6.0–6.9 ppm is caused by the existence of hydrogen on the surface of the benzene ring structure. In Figure 3b, there is only one single peak at −0.6 ppm, which indicates that only one phosphorus-containing compound exists. NMR and FTIR spectra show that DDP was successfully synthesized.

**Figure 4:**
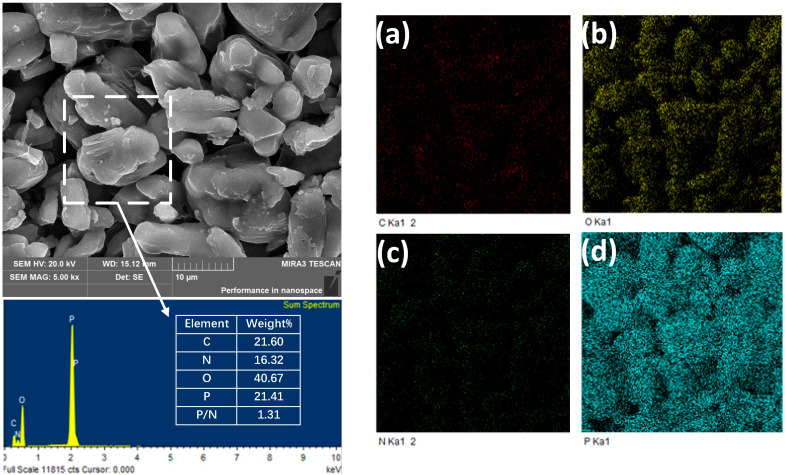
SEM images and EDS maps of DDP flame retardant (**a**) carbon, (**b**) oxygen, (**c**) nitrogen, and (**d**) phosphorus.

SEM-EDS analysis was used to investigate the morphology and element composition of DDP, and the SEM-EDS maps are presented in Figure 4. As shown in Figure 4, DDP causes obvious wrinkles on the surface of the APP owing to the adhesion of DDM, as supported by Zhao [19]. The contents of carbon, oxygen, and nitrogen elements, as well as phosphorus elements, in DDP (a, b, c, d) were distinguished through EDS. After the introduction of DDM, the carbon content of APP displayed an obvious improvement; the carbon element is evenly distributed on the APP surface, meaning that DDM is evenly distributed on the APP surface [20]. The above phenomena show that the DDP was synthesized as expected.

**Figure 5:**
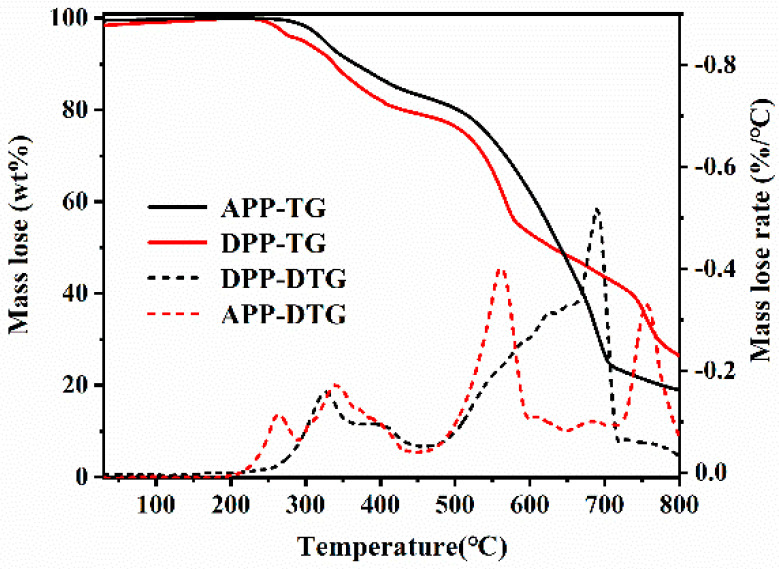
The mass loss and mass loss rate curves of DDP and APP at a heating rate of 10 °C min^−1^ under a nitrogen gas flow rate of 50 mL min^−1^.

Thermal decomposition processes of APP and DDP are analyzed by thermogravimetric analysis. As shown in Figure 5, DDP shows four pronounced decomposition processes at 200~290, 290~450, 450~600, and 720~800 °C, respectively, while APP shows two obvious decomposition processes at 250~450 and 450~720 °C. Besides, the char yields of APP and DDP are 28.1% and 44.6%, respectively, at 800 °C, indicating that DDP presents better char-forming ability than APP.

### 3.2. Thermal Behavior Analysis

**Figure 6:**
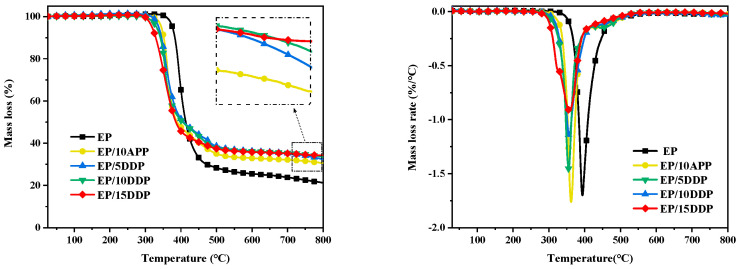
TG and DTG curves of flame-retarded EP blends at a heating rate of 10 °C min^−1^ in a nitrogen atmosphere.

TG test was used to evaluate the thermal behavior and char formation of flame retardant EP blends, and the curve is shown in Figure 6. As shown in Figure 6, all samples exhibit one degradation process, and the mass loss and mass loss rate of EP blends are lower compared with those of neat EP, indicating that the addition of DDP and APP weakens the thermolysis of EP blends.

The thermal parameters gained by the TG test are listed in Table 2. The *T*_5%_ and *T*_max_ values of neat EP are 375.8 °C and 391.8 °C, respectively, under nitrogen atmosphere. In contrast, the *T*_5%_ and *T*_max_ values of all EP blends greatly drop compared with neat EP, and the most obvious declines in *T*_5%_ (317.3 °C) and *T*_max_ (353.8 °C) were displayed by EP/15DDP, which refer to the C–N and P–O bonds fracturing earlier during decomposition than the C–C bond. The char yield of neat EP is 21.3% at 800 °C, whereas the char yield of EP blends notably increases. The Δ*W* value can evaluate the char-forming ability; the higher the Δ*W* values, the stronger its char-forming ability [21]. As shown in Table 2, the Δ*W* value of EP/10 DDP is higher than EP/10APP, indicating that the char-forming ability of APP was improved by grafting of DDM. Compared with neat EP, the char yield of EP/15DDP is increased by 60.6%. Besides, *W*_exp_ values of EP blends containing DDP are higher than the corresponding *W*_cal_, which suggests the stronger char-forming ability of DDP. Moreover, PMLR values of EP/5DDP, EP/10DDP, and EP/15DDP were decreased by 32.4%, 35%, and 34.2%, respectively, compared with neat EP, which reveals that the addition of DDP contributes to forming more residual weight at high temperatures [22].

### 3.3. DSC Analysis

**Figure 7:**
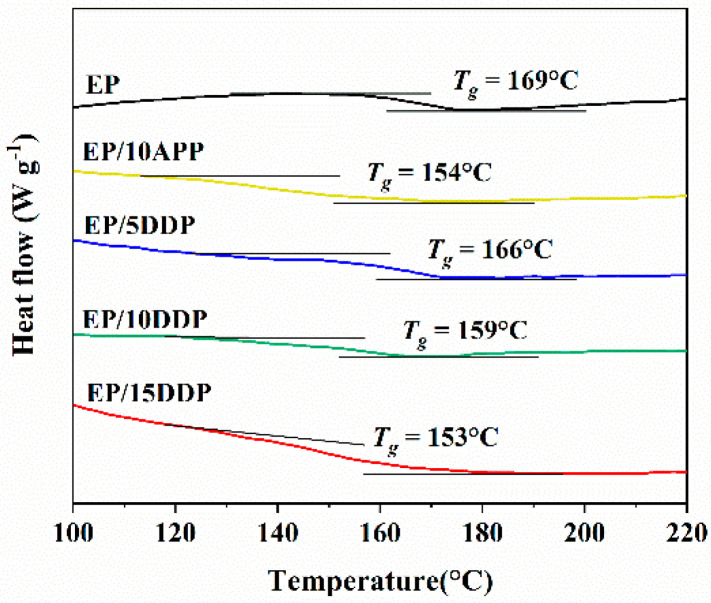
DSC curves of flame-retarded EP blends at a heating rate of 10 °C/min in nitrogen.

The glass transition temperature (*T*_g_) of flame-retarded epoxy resin was measured by DSC analysis, and the results are shown in Figure 7. It can be seen that *T*_g_ of neat EP is 169 °C, and the glass transition temperature of epoxy resin decreased after introducing APP and DDP. Moreover, the *T*_g_ value of EP/10DDP is 5 °C higher than EP/10APP, which indicates that the modification process weakens the negative influence of flame retardant additives on the glass transition temperature of epoxy resin [23]. This may be caused by the reaction between –NH_2_ on the surface of DPP and epoxy groups, indicating that DDP has more satisfying compatibility in EPs than APP [24].

### 3.4. LOI and UL94 Tests

**Figure 8:**
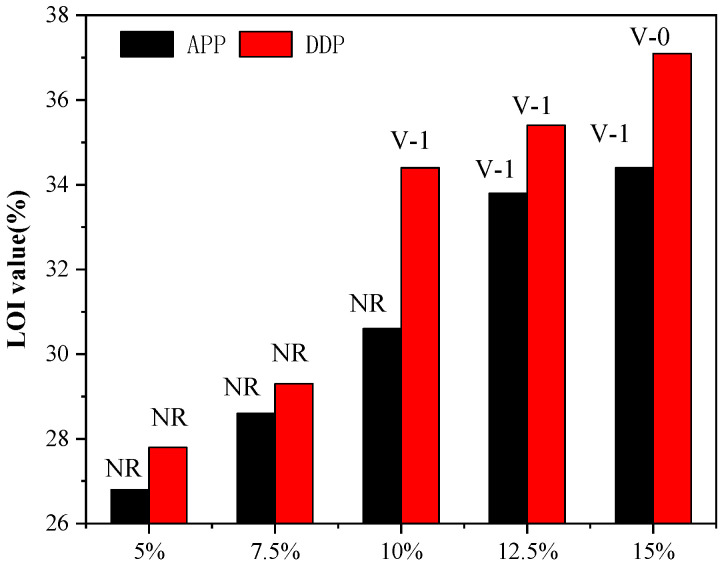
LOI value and UL94 rating of neat EP and EP blends.

LOI and UL94 tests were used to evaluate the flame retardant properties of neat EP and EP blends; the results are presented in Table 1 and Figure 8. The LOI value of neat EP is only 26.3% and cannot pass the UL94 test. With the introduction of APP and DDP, the LOI value and the UL94 rating of EP blends were increased to different degrees. It should be noted that EP/10DDP has a higher LOI value and UL94 rating than those of EP/10 APP, indicating that DDP has higher flame retardant efficiency than EP/10APP in EP blends. Moreover, EP/15DDP exhibited excellent fire resistance with an LOI value of 37.1% and passing UL94 V-0 rating.

**Figure 9:**
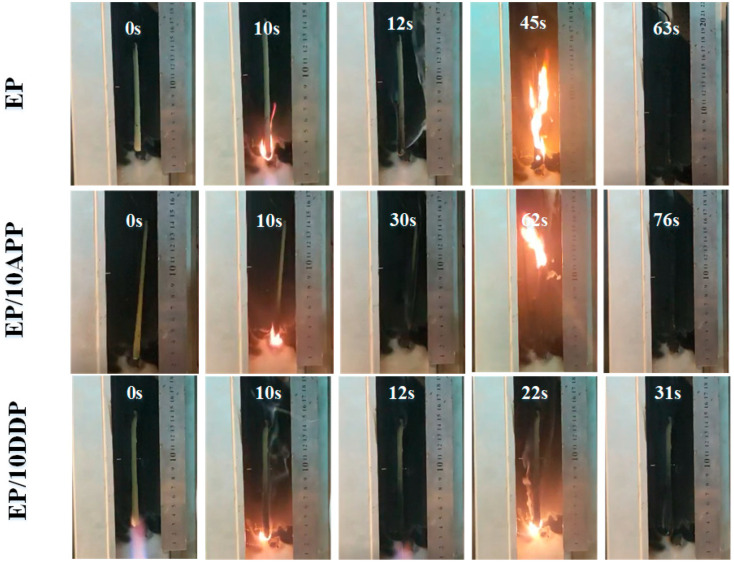
Digital photographs of the process of vertical burning tests of EP blends (from left to right, the initial state, the first ignition ends, the first flame goes out, the second ignition, and the second ignition ends).

In order to further study the flame-retarded effect of DDP in EP, the digital photos of the burning process in the UL94 test are shown in Figure 9. After the first flame application, the flame of EP/10DDP extinguishes rapidly, while the flame of EP/10 APP lasts for a long time. After the second flame application, the flames of neat EP and EP/10APP spread to the upper end rapidly, and the neat EP burns out. However, EP/10DDP gradually decreases to extinction after burning for some time, indicating that DDP in epoxy resin has a better inhibitory effect on flame propagation than APP.

### 3.5. Cone Calorimeter Test

**Figure 10:**
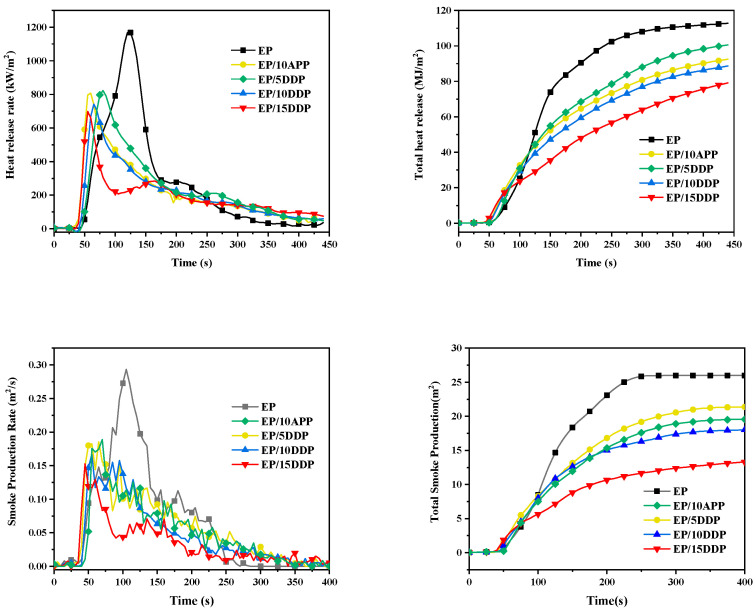
Cone calorimetric curves of flame-retarded EP blends: heat release rate (HRR), total heat release (THR), smoke production rate (SPR), and total smoke release (TSR).

In order to evaluate the combustion behavior of EP blends in a real fire condition, a cone calorimeter was carried out and the results are shown in Figure 10. Typical combustion parameters such as ignition time (TTI), peak heat release rate (PHRR) and total heat release (THR), peak smoke production rate (PSPR), total smoke release (TSR), and residual quality are summarized in Table 3.

As shown in Table 3, TTI of the EP blends slightly decrease with the increase in APP and DDP loadings compared with neat EP, which is owing to the lower decomposition temperature of APP and DDP, as supported by the TG test. As shown in Figure 10, neat EP burns intensely after ignition and reaches the combustion intensity at about 125 s, accompanied by a THR value of 111.8 MJ/m^2^ and a PHRR value of 1186.7 kW/m^2^. With the incorporation of flame retardants, the THR values of EP/10APP and EP/10DDP reduce to 98.6 MJ/m^2^ and 86.3 MJ/m^2^, respectively, and PHRR values reduce to 866.1 kW/m^2^ and 753.4 kW/m^2^, respectively, suggesting that both APP and DDP could improve the flame retardancy of epoxy resin. Compared with APP, the same addition of DDP imparts better flame retardancy to the EP, while the THR and PHRR values of the EP/15DDP sample reduce by 32.3% and 40.8%, respectively, compare with neat EP.

The smoke production rate (SPR) and total smoke release (TSR) curves of EP blends are shown in Figure 10. With the incorporation of APP and DDP, the initial smoke production time of EP blends is advanced, which is related to the early ignition time caused by the addition of APP and DDP. In the initial ignition stage, the PSR and TSR values of EP blends are lower than neat EP owing to the effective char layer that formed during combustion, which could inhibit the penetration of gas and smoke particles. Compared with neat EP, the PSPR and TSR values of EP/10APP are decreased by 35.5 and 17.7%, respectively, while EP/10DDP shows a 43.0% reduction in PSPR value and a 30.4% reduction in TSR value. The superior smoke suppression effect of DDP is ascribed to the good compatibility between DDP and epoxy resin that helps to form a denser coke layer against the transfer of heat and combustible gas, as supported by DSC analysis. Moreover, the same addition of DDP generates more residual weight after combustion compared with APP, and the increase in residual weight could contribute to the reduction in combustible gas and smoke [25]. Overall, DDP presents an obvious improvement of the flame retardant and smoke suppression effect compared with APP.

**Figure 11:**
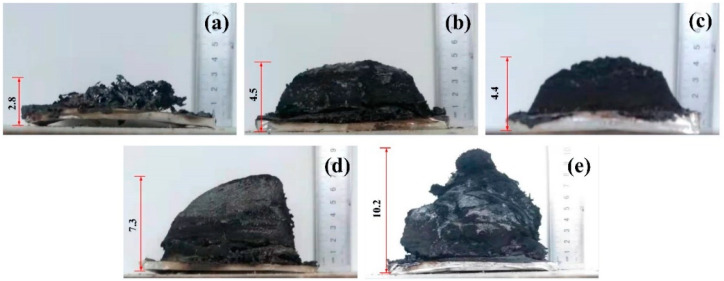
Digital photographs of the char residues obtained after the cone calorimeter test: (**a**) EP, (**b**) EP/10APP, (**c**) EP/5DDP, (**d**) EP/10DDP, and (**e**) EP/15DDP.

Figure 11 shows the char residues obtained after the cone calorimeter test. As shown in Figure 11, the char residue of neat EP has a height of 2.8 cm and its morphology is cracked, while flame-retarded EP blends generate a considerable volume of intumescent char layer. It can be found that the char layer formed by EP/10DDP is 2.8 cm higher than EP/10DDP, which indicates that DDP in EP blends has better char-forming ability. Moreover, the increase in char height is accompanied by the increase of DDP content, and the char height of the EP/15DDP sample reached 10.2 cm.

### 3.6. Char Residue Analysis

**Figure 12:**
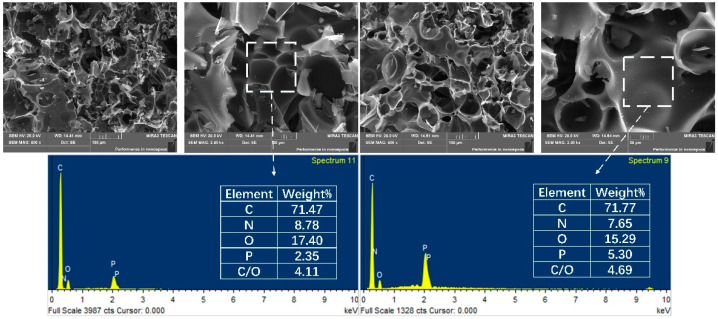
SEM images and EDS maps of the char residues of EP/10APP and EP/10DDP obtained after the cone calorimeter test.

SEM-EDS images of char residues of EP/10APP and EP/10DDP after the cone calorimeter test are presented in Figure 12. The EP/10APP char contains a large number of bubbles on the surface, which cannot effectively inhibit the diffusion of mass and heat. In contrast, EP/10DDP tends to form a more intact and continuous char layer with no cracks and fewer holes, thus exhibiting a superior barrier effect against fire. In addition, from the results of EDS mapping, it can be found that the content of phosphorus in EP/DDP char residue is higher than EP/10APP, which means more aromatization and crosslinking structures remained in the char layer [26]. Moreover, the C/O mass ratio of char residue is improved to 4.69 in EP/10DDP from that of 4.11 in EP/10APP, and a higher C/O mass ratio is beneficial to produce a char layer with better antioxidation property and denser structure [27].

**Figure 13:**
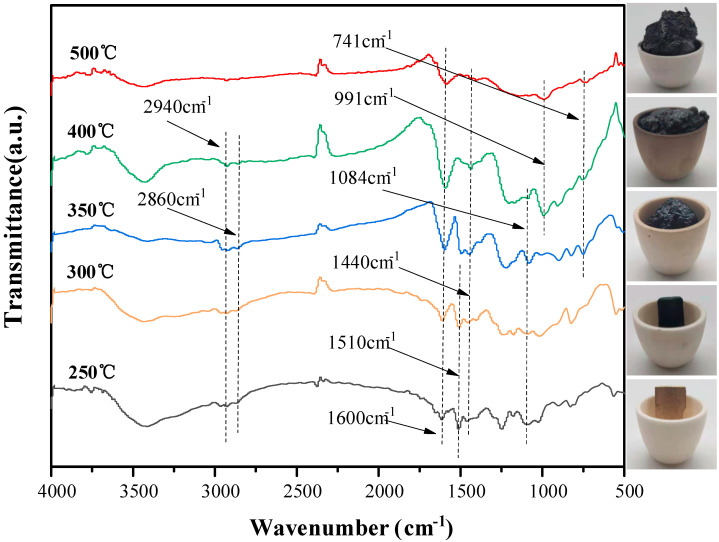
FTIR spectra of the char residues of EP/10DDP obtained after the cone calorimeter test.

In order to investigate the reason for the high effectiveness of flame retardant of DDP, the FTIR spectra and the digital photos of EP/10DDP at different temperatures are shown in Figure 13. The peak at 1510 cm^−1^ corresponding to the NH^3+^ structure of DDP decreased gradually and then disappeared at 400 °C, indicating the decomposition of the NH^3+^ groups. Moreover, new peaks of P–N–C groups are observed at 1084 cm^−1^ and 741 cm^−1^ at 350~400 °C [28,29], and the formation of P–N–C is beneficial to enhance the thermal stability of char [30]. Besides, the new peak at 991 cm^−1^ corresponding P–O–C groups appeared at 400 °C, indicating the formation of phosphorus-rich crosslinking structures during combustion [9]. The absorbing peaks of –CH_2_ and –CH_3_ at 2860 cm^−1^ and 2940 cm^−1^ gradually disappeared, while the peaks at 1600 cm^−1^ assigned to C=C groups become wider, suggesting the appearance of C=C structures. In addition, the peak at 1440 cm^−1^ is assigned to the absorption peak of C–C groups in the aromatic rings, indicating the formation of aromatic structures. Overall, the char rich in P–O–C, P–N–C, and C=C groups contributes to enhancing the heat insulation property and antioxidant ability of the samples, thus endowing the sample with superior flame retardancy and smoke suppression properties.

### 3.7. Tensile Test

**Figure 14:**
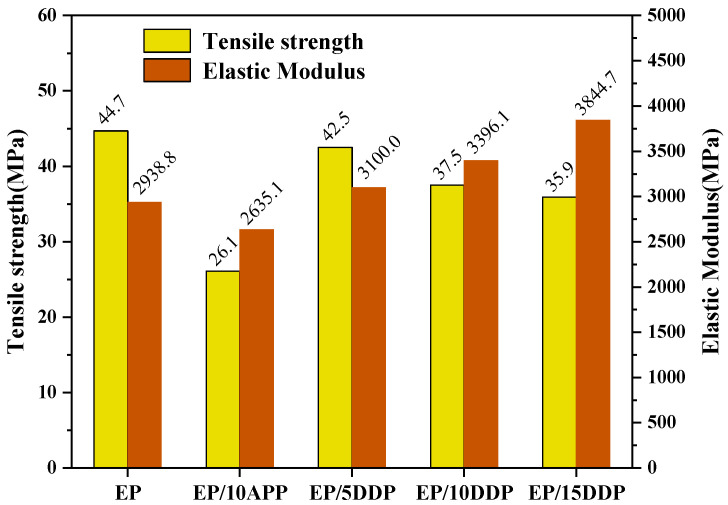
Tensile strength and elastic modulus of EP blends.

The mechanical property of flame retardant epoxy resin is shown in Figure 14. As shown in Figure 14, the tensile strength of EP blends gradually decreased with the introduction of flame retardant. It is obvious that the tensile strength of EP/10APP and EP/10DDP decreased by 41.6% and 16.1%, respectively, compared with neat EP, indicating that the same addition of DDP has a lesser negative influence on the EP blends compared with APP. In addition, the elastic modulus of EP blend with DDP is enhanced, while EP blends with APP show a downtrend, which is mainly related to the cross-linking reaction between amino groups on the surface of DDP and epoxy groups of flame-retardant epoxy resin, as supported by DSC analysis.

## 4. Conclusions

In this article, DDP was synthesized successfully by grafting DDM onto the surface of APP via the ion-exchange reaction. The chemical structure of DDP was carefully verified by FTIR, NMR, and SEM-EDS analyses. Then, the obtained DDP was incorporated into epoxy resin to prepare flame-retarded EP blends. The results of LOI and UL94 tests illustrate that the same addition of DDP exerts better flame-retardant efficiency in EP compared with APP. In particular, the EP blend containing 15 wt% DDP has an LOI value of 37.1% and passes the UL94 V-0 rating, while the EP blend containing 15 wt% APP has an LOI value of 34.4% and only passes the UL94 V-1 rating. Cone calorimeter test show that the incorporation of DDP greatly decreases the heat release and smoke production of EP, thus greatly reducing the fire hazard of EP. Compared with neat EP, the EP blend containing 10 wt% DDP shows a 22.8% reduction in THR value and a 30.4% reduction in TSR value. DSC analysis shows that DDP has a less negative influence on the glass transition temperature of EP than that of APP owing to the better compatibility between the EP matrix and DDP flame retardant. The tensile test illustrates that the same addition of DDP imparts higher tensile strength and Young’s modulus to EP compared with APP; the EP containing 15 wt% DDP has a tensile strength of 35.9 MPa and elastic modulus of 3844.7 MPa. Char residue analysis shows that the incorporation of DDP in EP contributes to forming more P–O–C, P–N–C, and C=C groups in the condensed phase that produce a char layer with excellent integrity and stability, thus endowing the EP with a satisfactory flame retardancy and smoke suppression properties. The DDP has superior flame-retardant efficiency and good compatibility in an epoxy matrix, which is expected in other polymeric materials.

## Figures and Tables

**Table 1:** Formulation and flammability tests of flame-retardant epoxy resin (mass fraction) %.

Samples	EP (wt%)	DDM (wt%)	APP (wt%)	DDP (wt%)	LOI (%)	UL 94 Rating
EP	80	20	0	0	26.3	No rating
EP/5APP	76	19	5	0	26.8	No rating
EP/7.5APP	74	18.5	7.5	0	28.6	No rating
EP/10APP	72	18	10	0	30.6	No rating
EP/12.5APP	70	17.5	12.5	0	33.8	V-1
EP/15APP	68	17	15	0	34.4	V-1
EP/5DDP	76	19	0	5	27.8	No rating
EP/7.5DDP	74	18.5	0	7.5	29.3	No rating
EP/10DDP	72	18	0	10	34.1	V-1
EP/12.5DDP	70	17.5	0	12.5	35.4	V-1
EP/15DDP	68	17	0	15	37.1	V-0

**Table 2:** Thermal parameters of the flame-retarded EP blends under a nitrogen atmosphere at a heating rate of 10 °C/min.

Samples	*T*_5%_ (°C)	*T*_max_ (°C)	PMLR (%/min)	*W*_exp_ (%)	*W*_cal_	Δ*W*
EP	375.8	391.8	1.68	21.3	--	--
EP/10APP	344.7	361.2	1.72	30.6	21.98	8.62
EP/5DDP	337.3	356.7	1.45	32.4	22.47	9.93
EP/10DDP	331.0	353.8	1.14	33.5	23.63	9.87
EP/15DDP	317.3	355.7	0.91	34.2	24.80	9.40

Note: *T*_5%_—initial decomposition temperature, *T*_max_—the temperature of maximum mass loss rate, PMLR—peak mass loss rate, Δ*W* = *W*_exp_ − *W*_cal_.

**Table 3:** Cone calorimeter data of the flame-retarded EP blends at a heat flux of 50 kw/m^2^.

Samples	TTI (s)	PHRR (kW/m^2^)	THR (MJ/m^2^)	PSPR (m^2^/s)	TSR (m^2^/m^2^)	Residue (%)
EP	39	1186.7	111.8	0.293	1460.7	6.8
EP/10APP	29	866.1	98.6	0.189	1202.2	23.3
EP/5DDP	26	826.9	90.2	0.185	1102.6	24.9
EP/10DDP	34	753.4	86.3	0.167	1016.6	28.8
EP/15DDP	33	702.4	75.7	0.153	759.0	34.0

## Data Availability

The data presented in this study are available upon request from the corresponding author.
